# ReStOre@Home: Feasibility study of a virtually delivered 12-week multidisciplinary rehabilitation programme for survivors of upper gastrointestinal (UGI) cancer - study protocol

**DOI:** 10.12688/hrbopenres.13185.2

**Published:** 2021-05-04

**Authors:** Linda O'Neill, Emer Guinan, Louise Brennan, Suzanne L. Doyle, Louise O'Connor, Grainne Sheill, Emily Smyth, Ciaran M. Fairman, Ricardo Segurado, Deirdre Connolly, Jacintha O'Sullivan, John V. Reynolds, Juliette Hussey

**Affiliations:** 1 Discipline of Physiotherapy, Trinity College Dublin, the University of Dublin, Dublin, Ireland; 2 Trinity St James's Cancer Institute, Dublin, Ireland; 3 School of Medicine, Trinity College Dublin, the University of Dublin, Dublin, Ireland; 4 School of Biological and Health Sciences, Technological University Dublin, Dublin, Ireland; 5 Department of Exercise Science, Arnold School of Public Health, University of South Carolina, Columbia, USA; 6 School of Public Health, Physiotherapy and Sports Sciences,, University College Dublin, Dublin, Ireland; 7 Discipline of Occupational Therapy, Trinity College Dublin, the University of Dublin, Dublin, Ireland; 8 Department of Surgery, Trinity Translational Medicine Institute, Trinity College Dublin, the University of Dublin and St James's Hospital Dublin, Dublin, Ireland

**Keywords:** Virtual delivery, multidisciplinary rehabilitation, upper gastrointestinal cancer, exercise, diet, education

## Abstract

**Background:** Exercise rehabilitation programmes, traditionally involving supervised exercise sessions, have had to rapidly adapt to virtual delivery in response to the coronavirus disease 2019 (COVID-19) pandemic to minimise patient contacts. In the absence of an effective vaccine, the pandemic is likely to persist in the medium term and during this time it is important that the feasibility and effectiveness of remote solutions is considered.  We have previously established the feasibility of the Rehabilitation Strategies following Oesophago-gastric Cancer (ReStOre) intervention - a face to face multidisciplinary rehabilitation programme for upper gastrointestinal (UGI) cancer survivors. This study will examine the feasibility of a virtually delivered 12-week multi-component ReStOre@Home programme.

**Methods:** This single arm feasibility study will recruit 12 patients who have completed curative treatment for oesophago-gastric cancer. Participants will complete the 12-week ReStOre@Home programme consisting of exercise (aerobic and resistance training), 1:1 dietary counselling and group education sessions through virtual delivery. Underpinned by the Medical Research Council (MRC) Framework, feasibility will be determined by recruitment rates, adherence, retention, incidents, and acceptability. Acceptability will be assessed qualitatively through post-intervention interview and the Telehealth Usability Questionnaire. Secondary outcomes will be assessed pre and post-intervention and will include measures of physical performance (cardiopulmonary exercise test, short physical performance battery, hand grip strength, Godin Leisure Time Questionnaire, and body composition), health related quality of life (European Organisation for Research and Treatment of Cancer Core Quality of Life Questionnaire (EORTC-QLQ-C30) and oesophago-gastric cancer specific subscale (EORTC-QLQ-OG25), fatigue (Multidimensional Fatigue Inventory (MFI-20), and venous blood samples will be collected for the UGI Cancer Survivorship Biobank.

**Discussion:** The ReStOre@Home feasibility study will provide important data regarding the amenability of a multidisciplinary programme designed for UGI cancer survivors to virtual delivery.

**Trial registration:** ClinicalTrials.gov NCT04603339 (26/10/2020)

## Introduction

We have previously established the safety, feasibility and initial efficacy of multidisciplinary rehabilitation in oesophago-gastric cancer survivorship, an understudied cohort of cancer survivors with significant nutritional, functional, and quality of life needs ^1– 3^. The ReStOre (Rehabilitation Strategies following Oesophago-gastric Cancer) feasibility study ^4, 5^ and pilot randomised controlled trial (RCT) ^6, 7^ demonstrated that a 12-week programme of supervised and homebased exercise, 1:1 dietary counselling, and health education could result in clinically significant improvements in cardiorespiratory fitness and physical and mental well-being without compromise to body composition in this nutritionally vulnerable cohort. Thus the ReStOre RCT is the first evidence-based model of rehabilitation in UGI cancer survivorship. The ReStOre II (Rehabilitation Strategies following Oesophagogastric and Hepatopancreaticobiliary Cancer) RCT now plans to further examine the effectiveness of the ReStOre programme by RCT in a larger cohort of upper-gastrointestinal (UGI) cancer survivors ^8^. However, due to the coronavirus disease 2019 (COVID-19) pandemic plans to commence recruitment to the ReStOre II RCT have been delayed until public health advice facilitates implementation of such activities.

The COVID-19 pandemic has changed all our lives and how we go about our activities of daily living, including how we exercise. Rehabilitation including exercise therapy is an important part of recovery from cancer ^9^, and efforts to continue these interventions are a priority despite COVID-19 ^10^. However, delivery of rehabilitative programmes has been greatly inhibited due to the pandemic. Current barriers to the implementation of cancer rehabilitation in Ireland and internationally include the need for vulnerable cohorts to cocoon, a reluctance amongst high risk cohorts to attend appointments in healthcare environments due to infection control fears, the need for physical distancing, public health recommendations to minimise use of public transport, and rolling restrictions ^11^. As a means of overcoming these barriers, remote delivery is an attractive alternative mode of providing much needed rehabilitative services to cancer survivors within the safety of their own homes ^12^. In recent years, the feasibility and efficacy of delivering rehabilitation virtually to patients living with and beyond cancer has been increasingly explored in exercise oncology research. Whilst initial results of trials are largely supportive of virtual delivery ^13, 14^, little is known regarding the feasibility of delivering multidisciplinary rehabilitation virtually to survivors of UGI cancers. Whilst video-conferencing provides an ideal vehicle for delivery of established rehabilitation programmes at this time, successful transition to a virtual model needs to be multidimensional, delivering all the essential components of the planned rehabilitative intervention including group exercise sessions, 1:1 dietary consultations, group education sessions and opportunity for group discussion. These contrasting modes of participant engagement and interaction requires rigorous evaluation to establish effectiveness and comparability to face-to-face models of care. Moreover, face to face programmes in cancer survivorship are advocated for their innate social value, whereby participants benefit hugely from the peer support gained from meeting and engaging with other cancer survivors, validating their role as experts in their condition ^7^. However, it is unknown if such social benefits may translate to a virtually delivered programme.

To this end, the COVID-19 pandemic presents an excellent opportunity to discover more about the potential of the virtual delivery of multidisciplinary rehabilitation to UGI cancer survivors. Although complex to implement given the multi-component nature of the programme, consultation with public and patient involvement (PPI) representatives indicates investigation of delivery of this programme virtually would be thoroughly welcomed by this patient cohort as the pandemic persists. Accordingly, we will explore this issue through the implementation of a sub-study to the planned ReStOre II RCT entitled ‘ReStOre@Home’ which will be underpinned by the Medical Research Council (MRC) Framework for evaluating complex interventions ^15^.

### Study aims

The overall aim of this work is to examine the feasibility of implementing a 12-week multidisciplinary rehabilitation programme consisting of aerobic and resistance exercise, dietary counselling, and education sessions delivered virtually via video-conferencing for survivors of UGI cancer.

Feasibility will be determined by the following outcomes;

i) Recruitment rate
ii) Adherence rate
iii) Acceptability of the programme
iv) Retention
v) Incidents

Secondary aims are:

- To examine the effect of the ReStOre@Home programme on physical functioning.
- To explore the effect of the ReStOre@Home programme on dietary quality and nutritional status.
- To examine the effects of the ReStOre@Home programme on patient reported outcomes including HRQOL, and fatigue.

## Methods

### Study design

**Figure 1.**
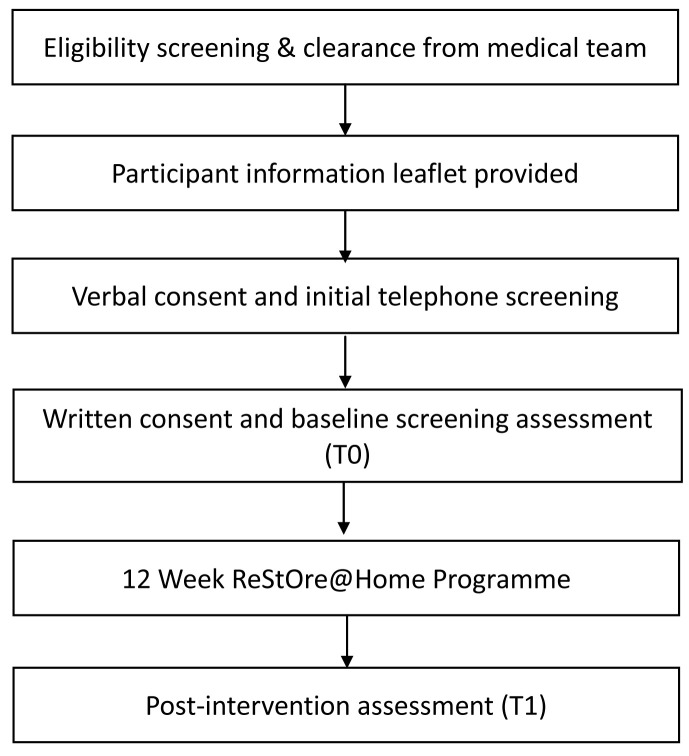
Study design.

ReStOre@Home will be implemented as a single arm, feasibility study. This feasibility work will be part of a series of work to complete a process evaluation of ReStOre@Home underpinned by the MRC framework for evaluating complex interventions ^16^. Table 1 describes the feasibility/piloting phase of the MRC Framework alongside the activities involved in this process evaluation ^15^. Ethical approval has been granted from the Tallaght University Hospital (TUH)/ St James’s Hospital (SJH) Ethics Committee. Any amendment to the protocol which may impact on the conduct of the study will be submitted as an amendment for approval to the ethics committees. The study will be performed according to the Declaration of Helsinki. The flow of participants through the study is depicted in Figure 1.

**Table 1.** Mapping activities to Medical Research Council (MRC) Framework.

2 Assessing feasibility and piloting methods			
2.1	Testing procedures for acceptability, compliance, and intervention delivery	i.	Testing procedures and intervention prescription previously determined feasible in pilot RCT work.
		ii.	Potential acceptability of telehealth intervention discussed with PPI representatives.
		iii.	Assess feasibility of delivering intervention via telehealth in terms of recruitment, retention, and usability through a pilot with 12 participants.
		iv.	Assess acceptability through qualitative interviews.
2.2	Estimating recruitment and retention	i.	Recruitment from a single, national cancer centre.
		ii.	Review of literature and engagement with trial methodology groups e.g. Health Research Board Trial Methodology Research Network (HRB-TMR) and MRC-NIHR Trials Methodology Research Partnership (TMRP) symposia and working groups to determine best practice for ongoing retention of participants.
2.3	Determining sample size	i.	Feasibility results may be used to inform sample size calculation of a future controlled trial.

### Study participants

ReStOre@Home will recruit 12 patients with a histological confirmed diagnosis of cancer of the oesophagus or stomach who have undergone surgery with curative intent. Participants must meet the following eligibility criteria:

- be ≥ three months post oesophagectomy, total gastrectomy
- with or without neo-adjuvant/adjuvant chemo/chemoradiotherapy with curative intent
- adjuvant therapy must be completed
- access to home broadband
- medical clearance to participate

Exclusion criteria are; ongoing serious post-operative morbidity, and evidence of active or recurrent disease. In addition those with any serious co-morbidity that would impact on exercise participation will be excluded including those with; uncontrolled hypertension (resting systolic blood pressure >180mmHg and/or diastolic >100mmHg), recent serious cardiovascular events (within 12 months) including, but not limited to cerebrovascular accident, and myocardial infarction, unstable cardiac, renal, lung, liver or other severe chronic disease, uncontrolled atrial fibrillation, and left ventricular function <50%.

Participants will be recruited from one hospital site, SJH Dublin, the National Centre for Oesophago-gastric Cancer in Ireland. Recruitment for ReStOre@Home will not occur at the same time as recruitment for the main ReStOre RCT. Participants will be identified at post-operative clinics and through institutional databases by their clinical team. Eligibility screening will be completed by the clinical team in conjunction with the research team at SJH. All participants will require medical clearance prior to enrolment. Participants will continue with all routine care as planned during their participation in the study. Potentially eligible patients will be informed about the study by a member of the research team in person or via telephone and will receive a participant information leaflet. Following a reflection period of 1 week, a researcher will telephone the patient to confirm their interest in participation.

As a consequence of the ongoing COVID-19 pandemic those interested in participating will be required to give initial consent verbally over telephone. Researchers will then schedule a baseline assessment which will be conducted in the Wellcome Trust-Health Research Board Clinical Research Facility (CRF) at SJH. In advance of the assessment as much information as possible will be collected via telephone interview e.g. background medical history, dietary interview and questionnaires will be provided in advance to minimise face to face contact. Written informed consent will be obtained during the baseline assessment (Extended data ^17^). If there are any findings at assessment which indicate that a person is unsafe to exercise, they will not proceed with the intervention phase of the study.

### Intervention

**Figure 2.**
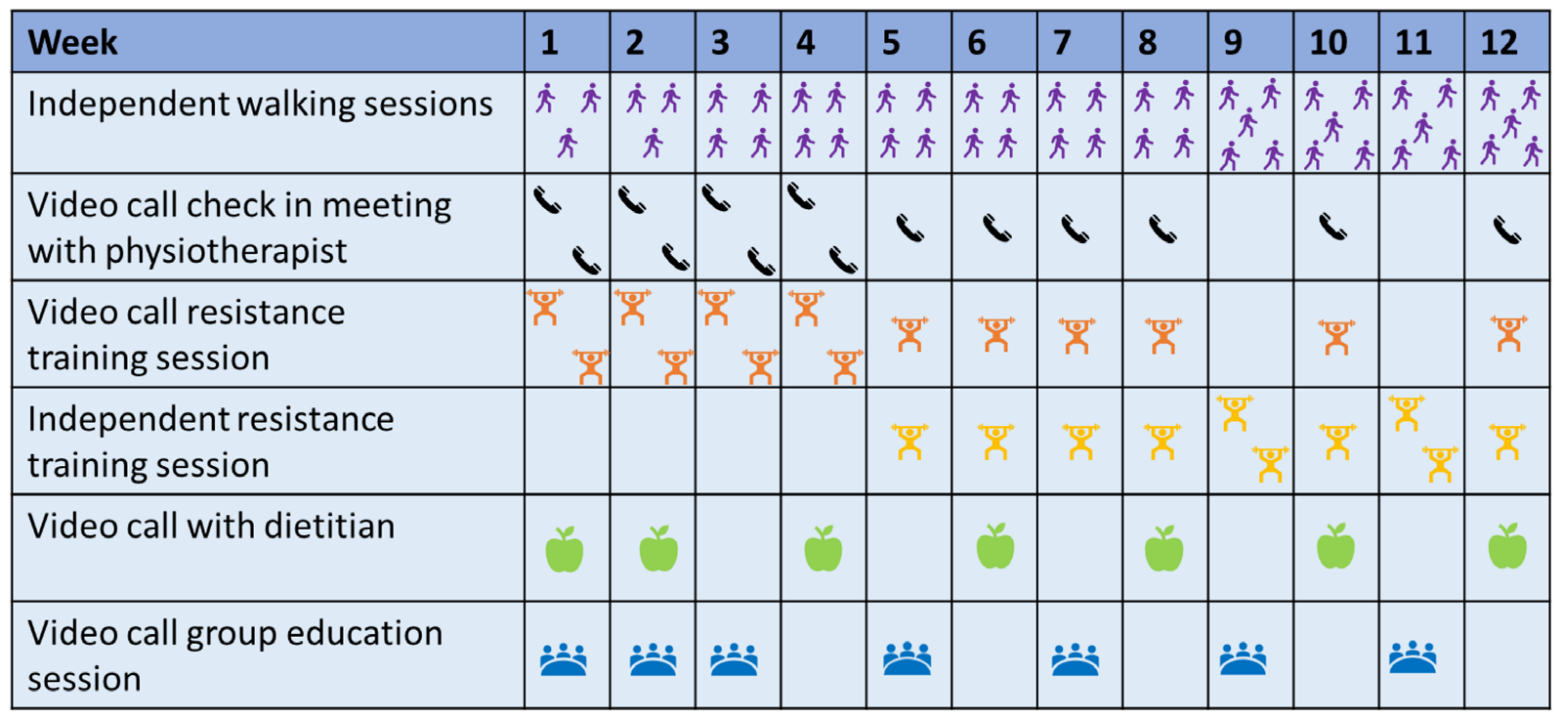
Frequency of ReStOre@Home Sessions.

The ReStOre@Home intervention will be delivered virtually through a video-conferencing platform and will follow a modified version of our established protocol for the ReStOre II RCT ^8^, the feasibility of which has been previously determined ^4, 6^. The ReStOre@Home programme comprises of three elements: exercise training, individualised dietetic counselling, and multidisciplinary education. The intervention is summarised in Figure 2. Coordination of the multicomponent virtual intervention will be overseen by the project manager, a physiotherapist experienced in the delivery of multimodal interventions. All video-conferencing sessions including the group education and resistance training sessions, and individual goal setting and dietetic counselling sessions will follow a defined schedule which will be provided to participants at the start of the intervention. To ensure the schedule is acceptable to each participant, group sessions will be planned for the same time each week and 1:1 sessions will be scheduled at times chosen by participants. Group education sessions will last a maximum of one hour; check-in meetings and group resistance training will last approximately 30 minutes.

**Figure 3.**
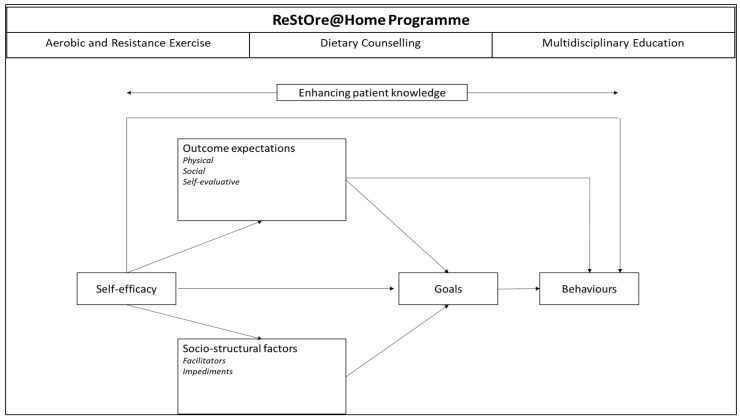
Social Cognitive Theory. *Figure 3 has been adapted with permission from Bandura A. Health promotion by social cognitive means. Health Educ Behav. 2004 Apr;31(2):143–64. doi: 10.1177/1090198104263660. PMID: 15090118 ^19^*.

ReStOre@Home will aim to give participants a greater sense of self-efficacy over their recovery from UGI cancer, to give them the belief that they can safely return to physical activity following their cancer journey, and promote lasting healthy lifestyle changes. This aim is grounded in Social Cognitive Theory (SCT) ^18, 19^ as it considers perceived self-efficacy as a key determinant of health behaviour change. Other core determinants of the model include; knowledge of health risks, outcome expectations, and perceived facilitators and impediments of behaviour ^19^. The design of the ReStOre@Home programme incorporates each of these core determinants ( Figure 3). Key to the programme is the goal to enhance self-efficacy amongst participants. This goal will be targeted through enhancing patient knowledge across the three components of the programme (exercise, dietary counselling, group education sessions), providing participants with education on the benefits of exercise, exercise safety, maintaining a stable body weight, and managing other symptoms such as fatigue. Outcome expectations will be derived through the setting of individualised exercise and dietary goals throughout the programme. The programme is also developed with perceived facilitators and impediments of physical activity in mind. Key facilitators of the programme will be the clear structure, and a motivated rehabilitation team. The multidisciplinary nature of the programme will also help address barriers to activity e.g. fear of weight loss, fatigue etc. to maximise adherence to the programme.

***Aerobic and resistance exercise training***. The exercise component will consist of a 12-week programme of aerobic and resistance programme. Unsupervised aerobic exercise in the form of walking will be prescribed as per the F.I.T.T (Frequency, intensity, type and time) principles outlined in the ReStOre II protocol ^8^, commencing at a low intensity (40–45% heart rate reserve (HRR)) and progressing to a moderate-vigorous intensity (65–85% HRR) ^8^. During the ReStOre@Home Programme all walking sessions will be monitored by the participant’s Polar Heart Rate Monitor (Polar M200) which will be provided. Participants will grant the research team access to their Polar Flow account to allow them to monitor their progress. The physiotherapist will organise a video-conference call check-in meeting with participants twice weekly for the first month, once weekly for the second month and once per fortnight for the final month of the programme. During this meeting the physiotherapist will perform a subjective assessment to check in on how the participant is feeling and will review with the participant how they are doing with the programme, explain their exercise prescription for the coming days/week, and set personal goals with the participant.

Resistance exercises will be performed as described in the ReStOre II RCT protocol ^8^. Participants will complete two sessions of resistance training per week for the duration of the programme, targeting major muscle groups. Participants will commence resistance training at a low intensity (16 repetition max (RM), one set x 12 repetitions) and progress to a higher intensity of 7RM (4 sets x 6 repetitions). Supervised resistance training sessions will be held in small online groups (maximum of 6) with the study physiotherapist via video-conference call. As per the ReStOre II protocol ^8^ there will be a gradual transition from supervised to independent training as the programme progress. All participants will be provided with the equipment necessary to complete the programme at home including free weights, an aerobic step, resistance bands and a polar heart rate monitor. Participants will log details of all their exercise sessions in a logbook.

***Dietary counselling***. One-to-one dietetic sessions will be delivered via video-conference calls during week 1, week 2 and fortnightly thereafter, or more frequently if required. Dietetic sessions will be delivered by a registered dietitian. As per the ReStOre II RCT protocol ^8^ the target for participants undertaking the ReStOre@Home programme will be to optimise dietary intake, ensuring adequate energy and micronutrient status, in alignment with international guidelines for cancer survivors ^20, 21^.

***Multidisciplinary education sessions***. Over the 12-week intervention participants will receive seven group education sessions via video-conference call which will be delivered by multidisciplinary team members including a doctor, dietitian, occupational therapist, and physiotherapist. Group size during the education component will be limited to six participants per session to optimise opportunities for peer to peer engagement. Education topics will include items of concern to UGI cancer survivors including; self-management, benefits of physical activity, and fatigue management.

### Outcomes

The ReStOre@Home study outcomes are listed in Table 2. The main assessment battery will be performed at baseline (T0), and post-intervention (T1).

**Table 2.** ReStOre@Home Study Outcomes.

Outcome	Instrument	Baseline	Post- intervention
		T0	T1
**Primary outcome**			
Feasibility	Recruitment rates		
	Adherence		
	Acceptability		
**Secondary outcomes**			
Aerobic Fitness	Cardiopulmonary Exercise Test	X	X
Functional performance	Short Physical Performance Battery (SPPB)	X	X
Muscle Strength	Hand grip strength (HGS)	X	X
	Leg Press 1-RM	X	X
Physical activity	Godin Leisure-Time Exercise Questionnaire	X	X
Body composition	Anthropometry	X	X
	Mid arm and waist circumference Bioimpedance Analysis	X X	X X
Dietary intake	Dietary interview	X	X
	Foodbook24	X	X
Nutrition-related symptoms	Gastrointestinal Symptom Rating Scale (GSRS)	X	X
	Simplified Nutritional Appetite Questionnaire (SNAQ)	X	X
Quality of Life	EORTC-QLQ-C30	X	X
Cancer specific quality of Life	EORTC-QLQ-OG25 (oesophago-gastric cancer)	X	X
Fatigue	Multidimensional Fatigue Inventory (MFI-20)	X	X
Qualitative approach	Semi –structured interviews		X
Other			
Sociodemographic details	Participant self-report	X	
Medical/Cancer history	Medical records	X	
Incidents	Reports of patients/research personnel	X	X
Satisfaction with Telehealth	Telehealth Usability Questionnaire (TUQ)		X
Biobank samples	Blood samples	X	X

***Primary outcome – feasibility***. This study will focus on the feasibility/ piloting phase of the MRC framework for process evaluation ^16^. Feasibility of the ReStOre@Home intervention will be described in terms of recruitment rates, adherence, retention, acceptability of the programme and incidents. Recruitment rate will be defined as the percentage of eligible study population whom consent to participation. In line with the ReStOre II trial protocol ^8^, adherence will be recorded according to a comprehensive battery of outcomes including number of completed sessions, permanent treatment discontinuation, treatment interruption, dose modification, early session termination, and pre-treatment intensity modification ( Table 3), consistent with recommended practice for clinical exercise trials ^22^. A number of sources will be used to calculate adherence including; participants polar heart rate data, participants logbook of exercise completed, and the physiotherapist’s records of supervised sessions. Retention will be defined as the percentage of enrolled participants completing the post-intervention assessment. Acceptability of the intervention will be determined through the use of qualitative interviews and completion of the Telehealth Usability Questionnaire (TUQ) ^23^ post-intervention. Incidents will be recorded throughout the study period. Feasibility to proceed to a definitive trial of ReStOre@Home will be determined by considering all the above factors, and the specific achievement of the following criteria: ≥50% of eligible patients recruited; mean of ≥80% adherence to supervised exercise sessions and ≥70% adherence to unsupervised sessions; ≥83% attendance at T1 assessment.

**Table 3.** Exercise Adherence variables.

Variable	Definition
Total number of supervised sessions attended	Total number of scheduled programme sessions attended on video call.
Total number of unsupervised sessions completed	Total number of unsupervised sessions reported in exercise diary as complete
Total number of compliant aerobic sessions completed	Total number of aerobic sessions where prescribed aerobic exercise dosage was achieved
Total number of compliant resistance sessions	Total number of resistance sessions where prescribed resistance training dosage was achieved
Permanent treatment discontinuation	Permanent discontinuation of the ReStOre@Home programme before week 12
Treatment interruption	Missing at least three consecutive ReStOre@Home supervised resistance training sessions
Dose modification	Number of videocall supervised sessions requiring exercise dose modification
Early session termination	Number of videocall supervised sessions requiring early session termination
Pre-treatment intensity modification	Number of videocall supervised sessions requiring modification because of a pre- exercise screening indication.

Feasibility will be further examined using a qualitative approach, wherein the acceptability of delivering the programme virtually will be explored along with participant’s perceptions of the impact of the ReStOre@Home programme on their physical and mental well-being. Data will be collected through semi-structured individual interviews immediately post-intervention (T1) by a researcher experienced in qualitative methods. Interviews will be held via telephone/video-conference call and will be recorded. The discussion guide (Extended data ^17^) will explore recommendations for future delivery of the programme through telehealth and the impact of the programme on overall health and wellbeing. Interview recordings will be transcribed and analysed using thematic analysis ^24^.

***Secondary outcomes***. Secondary outcomes will investigate the preliminary efficacy of the ReStOre@Home intervention, by examining the impact of the intervention on physical functioning, dietary adequacy and nutritional status, health related quality of life, and fatigue. These outcomes are exploratory only as the sample size is not sufficient to demonstrate treatment effect. The feasibility of utilising these measures in this cohort was previously established in the ReStOre I feasibility study and pilot RCT ^4, 6^. Physical functioning will be examined using a suite of validated measures examining aerobic fitness, functional performance, muscle strength, physical activity and body composition. Aerobic fitness will be determined by Cardiopulmonary Exercise Test (CPET). The CPET procedure will be performed as outlined in the ReStOre II protocol ^8^. An antibacterial/antiviral filter will be installed in the CPET circuit to minimise infection risk by reducing the amount of droplet aerosol dispersion in the air mitigating the contamination of the environment during testing. Functional performance will be captured using the Short Physical Performance Battery ^25^. Muscle strength will be assessed by handheld dynamometry and a 1-RM leg press test. Hand grip strength (HGS) provides a measure of hand and forearm strength and is found to correlate well with overall muscle strength and physical function ^26^. The 1-RM leg-press test will be performed as per the ReStOre II trial protocol ^8^. Physical activity levels will be measured by the Godin Leisure-Time Exercise Questionnaire, a validated tool for determining physical activity levels in cancer survivors ^27^. Weight (kilogrammes (kg)) and height (centimetres (cm)) will be recorded by standard methods and body mass index (BMI) will be calculated as weight (kg)/ height (metres (m ^2^)). Mid-arm muscle circumference and waist circumference will be measured in centimetres with a flexible measuring tape. Measurements will be taken in duplicate and averaged for data entry. Bioimpedance analysis (BIA) will be performed using Seca mBCA 515 (Seca, Hamburg, Germany).

Dietary adequacy and nutrition related symptoms will be assessed by the trial dietitian at T0 and T1 using a structured dietary interview. In addition participants will also complete Foodbook 24 ^28^, the Gastrointestinal Symptom Rating Scale (GSRS) ^29^ and the Simplified Nutritional Appetite Questionnaire (SNAQ) ^30^. Health Related Quality of Life (HRQOL) will be determined by the European Organisation for Research and Treatment of Cancer Quality of Life Questionnaire (EORTC-QLQ-C30 version 3.0) ^31^ and the oesophago-gastric cancer specific subscale (EORTC-QLQ-OG25). Fatigue will be assessed using the Multidimensional Fatigue Inventory (MFI-20).

***Biosample collection***. As per the ReStOre II RCT ^8^ participants will be invited to consent to donating samples to the Upper Gastrointestinal Cancer Survivorship Biobank (Extended data ^17^). Serum, plasma, and whole blood samples will be collected from consenting participants at T0 and T1. Samples will be processed and stored at -80°C at the Trinity Translational Medicine Institute, St James’s Hospital, Dublin 8 for future analyses to explore the impact of multidisciplinary rehabilitation in survivorship on key biomarkers.

### Safety

All incidents will be recorded, and serious incidents will be reported to the research ethics committee. Prior to baseline testing, all participants will require medical approval confirming their suitability for participation. Weight loss is a concern for UGI cancer survivors, and accordingly the study dietitian will monitor weight closely during the ReStOre@Home programme.

In light of the current pandemic additional measures to enhance safety will be implemented. All participants will be screened for signs and symptoms of COVID-19 via telephone by the research team the day before their assessments in the CRF at SJH. Participants will be screened again on the day of their assessment upon arrival at the CRF to confirm the participant and all individuals in their household are free from symptoms of COVID-19. All research staff will follow the COVID-19 National Protocol for workers and will not present themselves for work if symptomatic. Research staff will be fully equipped with alcohol hand gel, PPE and cleaning products and will receive training in how to use all correctly. As much of the assessment battery will be performed over the phone in advance of study assessments to minimise face to face contact time. Questionnaires will be provided via post to participants in advance of their assessment to further reduce face to face contact time. During the assessment in the CRF participants will be required to don a mask and clean their hands upon arrival and research staff will don appropriate PPE including a facemask, and goggles, and maintain physical distancing as much as possible. As the intervention will be delivered completely in participants homes, participants will be provided with the ReStOre@Home Exercising at Home Advice Sheet (Extended data ^17^) educating them on normal and abnormal responses to exercise and what they should do if they experience an abnormal response.

### Statistical considerations

***Sample size calculation***. A sample of 12 participants will be recruited to determine the feasibility of the ReStOre@Home programme. This is based on the recommendations of Julious *et al*. ^32^ whom recommend a minimum sample size of 12 per group as a rule of thumb and justifies this based on rationale about feasibility and precision about the mean and variance ^33^, in order to inform future quantitative studies. Similar sample sizes have been utilised in other rehabilitation trials in cancer survivorship ^4, 5, 34– 37^.

***Data management and analysis***. The Data Management Plan (Extended data ^18^) will outline how research data will be handled during and after the project. The data management plan is a live document and will be reviewed regularly throughout the study. Source documents for this study will include hospital records, procedure reports and data collection forms. Outcome assessments will be recorded in a paper-based case report form. Data from the case report form will then be entered into a password protected computer data repository. Data validation will be used to avoid erroneous data entry. All participants will be allocated a unique study code. The key to the study code will be stored securely and separately. All paper records will be stored in locked filing cabinets, in a locked office in a restricted access building with swipe access. Electronic records will be stored on password protected encrypted devices. Upon completion of the trial an anonymised data set will be deposited on a secure online repository in line with open access publication requirements.

Quantitative data analysis will be performed using IBM SPSS Statistics 26 software, employing statistical best practice. An inspection of patient characteristics at baseline will be carried out. Summary statistics for continuous variables (means and standard deviations or median and ranges as appropriate) and categorical variables (counts and proportions) will be presented. A qualitative descriptive approach ^38^ will be taken to the analysis of qualitative data. Braun and Clarke’s ^24^ 6 stage approach to thematic analysis will be used to analyse all data collected by a team of researchers using nVivo 12 (QSR International, Australia).

### Trial management and governance

The management of this feasibility study will be overseen by the ReStOre II trial management groups; a Trial management Group (TMG), Trial Steering Committee (TSC) and an Independent Data Monitoring Committee (IDMC).

### Dissemination

The results of the ReStOre@Home feasibility study will be disseminated via peer-reviewed publications and conference presentations. Upon completion of the trial an anonymised data set will be deposited on a secure online repository in line with open access publication requirements.

### Study status

Recruitment will begin in Winter 2020.

### Ethical statement

Ethical approval has been granted by the TUH/SJH Research Ethics Committee (REC: 2020-07 List- Amendment (23)). Any modifications to this planned protocol will be reported to the ethics committee.

The study was registered with ClinicalTrials.gov on 26 ^th^ October 2020 ( NCT04603339).

## Discussion

In the absence of an effective vaccine, the COVID-19 pandemic is likely to persist into the medium term. Whilst society continues to grapple with living with COVID-19, implementation of much needed rehabilitative programmes to people living with and beyond cancer in a face to face manner will continue to be extremely challenging due to public health restrictions and the valid fear experienced by vulnerable cancer survivors of attending face to face appointments in health care environments. To this end, never has the virtual delivery of rehabilitation to cancer survivors within their homes had such potential.

Implementing a complex multicomponent intervention virtually will not be without its challenges. Whilst existing evidence supports the implementation of single component virtual programmes such as online exercise classes, there is however emerging evidence to support the virtual delivery of other multi-component rehabilitation programmes in chronic disease management. Of note virtually delivered pulmonary rehabilitation programmes consisting of exercise, education, and self-management support has been found to be feasible, safe and result in equivalent clinical gains in comparison to face to face delivery ^39^ however the application of these results to other multimodal rehabilitation programmes, particularly those combining different individual interventions, is unknown and requires investigation. Successful implementation of the ReStOre@Home feasibility study would indicate the need to continue the process evaluation of the ReStOre@Home programme by RCT to assess its effectiveness. In the future, ReStOre@Home may potentially provide a viable alternative template for delivery of the ReStOre II programme to vulnerable patients who are cocooning or shielding in their homes, or those who would be otherwise unable to participate due to time constraints, or travel restrictions.

## Data availability

### Underlying data

No data are associated with article.

### Extended data

Open Science Framework: ReStOre@Home: Feasibility study of a virtually delivered 12-week multidisciplinary rehabilitation programme for survivors of upper gastrointestinal (UGI) cancer: Study Protocol https://doi.org/10.17605/OSF.IO/EYV3M

This project contains the following extended data:

- ReStOre@Home Interview Guide Version 1.pdf (Focus group/interview guide)
- ReStOre@Home Interview Guide Version 2.pdf (Focus group/interview guide)
- ReStOre@Home SJH Consent Form Version 1.pdf (Consent form)
- 200908 PIL_ICF_V1_Upper GI Surviorship Biobank ReStOre@Home.pdf (Biobank PIL and Consent Form)
- Data Management Plan (DMP) Version 1 RESTORE@HOME.pdf (Data Management Plan)
- ReStOre@Home Exercising at Home Advice Sheet.pdf (ReStOre@Home Exercising at Home Advice Sheet)
- 200626 ReStOre@Home Participant Logbook Version 1.pdf (Exercise session logbook)

### Reporting guidelines

Open Science Framework: SPIRIT checklist for ‘ReStOre@Home: Feasibility study of a virtually delivered 12-week multidisciplinary rehabilitation programme for survivors of upper gastrointestinal (UGI) cancer - study protocol’ https://doi.org/10.17605/OSF.IO/EYV3M

Data are available under the terms of the Creative Commons Attribution 1.0 Universal license (CC0 1.0).
