# Supplementary material for: ReStOre@Home: Feasibility study of a virtually delivered 12-week multidisciplinary rehabilitation programme for survivors of upper gastrointestinal (UGI) cancer - study protocol
Source: HRB Open Res. 2021 May 4;3:86. Originally published 2020 Nov 25. [Version 2] doi: 10.12688/hrbopenres.13185.2 (PMC8127012; doi:10.12688/hrbopenres.13185.2)
Supplement: Peer review history

# Peer review: Reviewer response for version 2

**DOI:** 10.21956/hrbopenres.14465.r29359

**Published:** 2021-05-14

**Authors:** Linda Denehy, Lara Edbrooke

**Affiliations:** 1 Melbourne School of Health Sciences, University of Melbourne, Melbourne, Australia; 2 University of Melbourne, Melbourne, Australia

We only had 1 outstanding question regarding an addition that has been made to the manuscript: “If there are any findings at assessment which indicate that a person is unsafe to exercise, they will not proceed with the intervention phase of the study.”

 Are the authors meaning the patient will be withdrawn from the study?

Is the study design appropriate for the research question?

Partly

Is the rationale for, and objectives of, the study clearly described?

Yes

Are sufficient details of the methods provided to allow replication by others?

Partly

Are the datasets clearly presented in a useable and accessible format?

Not applicable

Reviewer Expertise:

My research is focused on physiotherapy rehabilitation in acute care settings including critical care, pre and post surgery and oncology.

We confirm that we have read this submission and believe that we have an appropriate level of expertise to confirm that it is of an acceptable scientific standard.

# Peer review: Reviewer response for version 2

**DOI:** 10.21956/hrbopenres.14465.r29360

**Published:** 2021-05-13

**Authors:** Cynthia Forbes

**Affiliations:** 1 Wolfson Palliative Care Research Centre, Institute of Clinical and Applied Health Research, University of Hull, Hull, UK

No further comments. Reponses from authors reasonable and well done.

Is the study design appropriate for the research question?

Partly

Is the rationale for, and objectives of, the study clearly described?

Yes

Are sufficient details of the methods provided to allow replication by others?

Yes

Are the datasets clearly presented in a useable and accessible format?

Not applicable

Reviewer Expertise:

Cancer rehabilitation, digital technology.

I confirm that I have read this submission and believe that I have an appropriate level of expertise to confirm that it is of an acceptable scientific standard.

# Peer review: Reviewer response for version 1

**DOI:** 10.21956/hrbopenres.14318.r29019

**Published:** 2021-03-22

**Authors:** Cynthia Forbes

**Affiliations:** 1 Wolfson Palliative Care Research Centre, Institute of Clinical and Applied Health Research, University of Hull, Hull, UK

This article describes a study protocol for the modified version of a previously planned feasibility RCT to deliver a multi-component rehabilitation programme to upper GI cancer survivors. Feasibility for the in-person programme has been conducted and a larger trial was planned, now on hold due to the pandemic. In order to explore remote delivery, the authors have modified the protocol for video conference delivery.

 Comments

 A few question arose as I was reading, some of which were answered by the previous protocol.

 Is there a minimum and maximum time for the counselling, education, and exercise sessions?

 Study design: I say partly because the sample is too small to really estimate SD for a sample size calculation. Many sources call for minimum 30 participants for this kind of study. 12 may even be too few to reach saturation for the qualitative follow-up. Understandably, this may be a difficult population to recruit but I think this needs to be very clear. Subsequently, the secondary outcomes should be highlighted more so as exploratory.

 Do all people start the programme at the same time? With only 12, this may be feasible but I wondered how you would schedule these sessions.

 You mention having smaller groups for certain sessions. Will the groups always be the same people or will it be different each time?

 Access to broadband is essential but how will you deal with different levels of competence and grades of equipment? Will you exclude based on equipment availability, i.e. if someone doesn’t have a webcam, can they participate in sessions with a smartphone?

Is the study design appropriate for the research question?

Partly

Is the rationale for, and objectives of, the study clearly described?

Yes

Are sufficient details of the methods provided to allow replication by others?

Yes

Are the datasets clearly presented in a useable and accessible format?

Not applicable

Reviewer Expertise:

Cancer rehabilitation, digital technology.

I confirm that I have read this submission and believe that I have an appropriate level of expertise to confirm that it is of an acceptable scientific standard, however I have significant reservations, as outlined above.

# Response

**Published:** 2021-04-11

**Authors:** Linda O'Neill

**Affiliations:** Trinity University, Ireland

Dear Professor Forbes, 

 On behalf of the authorship I wish to thank you for taking the time to review our manuscript and for your constructive feedback. Please see below our responses to your comments.

**1. A few questions arose as I was reading, some of which were answered by the previous protocol.**

****

**2. Is there a minimum and maximum time for the counselling, education, and exercise sessions?**

*Response:*

 Thank you for your helpful feedback.

 Group education sessions will be a maximum of one hour. Group resistance training sessions will last approx. 30-40 minutes. 1:1 phone calls will last 30 minutes, apart from the first call which may be longer as participants may have additional questions.

 The following statement has been added to the ‘Intervention’ section of the manuscript:

 “Group education sessions will last a maximum of one hour; check-in meetings and group resistance training will last approximately 30 minutes.”

**3. Study design: I say partly because the sample is too small to really estimate SD for a sample size calculation. Many sources call for minimum 30 participants for this kind of study. 12 may even be too few to reach saturation for the qualitative follow-up. Understandably, this may be a difficult population to recruit but I think this needs to be very clear. Subsequently, the secondary outcomes should be highlighted more so as exploratory.**

*Response:*

 Thank you for this feedback. To reinforce that secondary outcomes are exploratory only, the following statement has been included in the ‘Outcomes’ section:

 “These outcomes are exploratory only as the sample size is not sufficient to demonstrate treatment effect.”

 We have carefully considered your comments regarding the sample size. We believe a sample size of 12 will provide sufficient data for the qualitative interviews. In accordance with recommendations from *Fusch et al*., we will apply specific measures to ensure the best chance of reaching data saturation: use of saturation grid, two-party coding of transcripts, and suitably-designed interview guides. We understand that, will well-conducted interview, data saturation can be achieved with 12 participants.

**4. Do all people start the programme at the same time? With only 12, this may be feasible but I wondered how you would schedule these sessions.**

*Response:*

 We aim to have two groups of six participants. Depending on recruitment rates, these two groups may be run at the same time or one after another; either way, they will operate separately to ensure that the groups of six have the opportunity to develop familiarity and social connections.

 5 **. You mention having smaller groups for certain sessions. Will the groups always be the same people or will it be different each time?**

*Response:*

 The groups of six will be the same participants each time.

**6. Access to broadband is essential but how will you deal with different levels of competence and grades of equipment? Will you exclude based on equipment availability, i.e. if someone doesn’t have a webcam, can they participate in sessions with a smartphone?**

*Response:*

 Participants will be welcome to join the online sessions on any device which has a webcam (desktop, laptop, tablet, smartphone). We will have opportunities to ensure they are familiar with the technology and provide tutorials or explanations at recruitment, assessment and during check-in calls. We will also provide each participant with ‘how-to’ information leaflets for the video-conferencing software and for the heart rate monitor.

 We hope that you find our response to your comments satisfactory and look forward to hearing from you. 

 Kind regards,

 Dr Linda O'Neill

**References**

 Fusch P, Ness L. Are we there yet? Data saturation in qualitative research. Qual Rep. 2015;20(9):1409-1416. doi:10.1177/1049732311401424

# Peer review: Reviewer response for version 1

**DOI:** 10.21956/hrbopenres.14318.r28448

**Published:** 2021-02-01

**Authors:** Linda Denehy, Lara Edbrooke

**Affiliations:** 1 University of Melbourne, Melbourne, Australia; 2 Melbourne School of Health Sciences, University of Melbourne, Melbourne, Australia

Thank you for the opportunity to review this well written protocol paper involving 12 weeks of 'virtual' rehabilitation for patients following treatment for oesophago-gastric cancer. This work is a sub study of an ongoing randomised controlled trial the ReStOre II RCT (NCT03958019) providing rehabilitation face to face for which the protocol is already published. The virtual program aims to establish the feasibility and safety of multi-disciplinary rehabilitation, including exercise, nutrition support and education, delivered entirely remotely.

 Given the impact the COVID-19 pandemic has had on traditional face-to-face models of rehabilitation delivery and taking into account rural or remote survivors who have limited access to services, work in this area is timely and of critical importance. 

 The protocol is well written and clear and generally reproducible. Following review of the manuscript we have the following questions and comments for the authors: 

1. Despite the justification references used for the sample size, I am not convinced that n=12 would provide enough patients to be representative of the target study population for using telehealth rehabilitation methods. The authors should remove the statement on page 9 that….’similar sample sizes have been used in in other rehabilitation trials in cancer’ This is not a scientific justification of sample size. Thabane *et al.* 2010 in A tutorial on pilot studies: what, why and how provide a simple confidence interval approach to estimating samples to establish feasibility ^1^. Additionally, I don’t believe that 12 patients will provide enough information on efficacy and variance estimates for the battery of outcome measures to estimate treatment effects in future trials and may lead to biased or unrealistic estimates. Further, the authors should keep in mind the impact that the COVID-19 pandemic could potentially have on the outcomes collected, including CPET, physical activity levels and health-related quality of life and caution against the use of these data for powering future trials.
2. This is a sub study of the larger RESTORE RCT. It is not documented how recruitment will work alongside the larger RCT at the one hospital site? This is important to ensure that bias is not introduced if patients have a choice or are not sequentially offered to participate in this smaller virtual sub study. Please add.
3. What cut-points will be used for the proportion of eligible patients recruited, adherence to intervention sessions and outcome assessments to guide whether implementing the intervention is feasible? Please add to aims/hypotheses.
4. If the baseline SPPB indicates balance impairments will participants still be prescribed outdoor walking or is loan of a stationary cycle for aerobic exercise possible? Please add to method.
5. Will participants be provided with equipment (bike/treadmill) in the event that walking outdoors is limited by government COVID-19 restrictions? Our recent experience is that participants may be wary of walking outdoors given the current situation. A suggestion.
6. The authors state one of the key aims of the programme is to increase participants’ self-efficacy over their recovery – the authors could consider including a patient-reported self-efficacy questionnaire to assess this. It would also strengthen the study to have an objective measure of physical activity using accelerometry, in addition to the use of the Godin Leisure Time Exercise questionnaire. A suggestion.
7. The authors state and Figure 2 highlights that this is a complex intervention. Will weekly video calls from the dietitian, the physiotherapist and group education sessions be co-ordinated/performed in conjunction to reduce the burden on participants of multiple weekly contacts? Please ad to method.
8. It is not clear (or may have missed it) if all outcome measures are being performed at the hospital? Clearly CPET would be but for others there is potential to undertake these remotely as well? This should be made clear.

Is the study design appropriate for the research question?

Partly

Is the rationale for, and objectives of, the study clearly described?

Yes

Are sufficient details of the methods provided to allow replication by others?

Partly

Are the datasets clearly presented in a useable and accessible format?

Not applicable

Reviewer Expertise:

My research is focused on physiotherapy rehabilitation in acute care settings including critical care, pre and post surgery and oncology.

We confirm that we have read this submission and believe that we have an appropriate level of expertise to confirm that it is of an acceptable scientific standard, however we have significant reservations, as outlined above.

## References

[1] : A tutorial on pilot studies: the what, why and how. *BMC Med Res Methodol*.2010;10: 10.1186/1471-2288-10-1 1 10.1186/1471-2288-10-1 20053272PMC2824145

# Response

**Published:** 2021-04-11

**Authors:** Linda O'Neill

**Affiliations:** Trinity University, Ireland

Dear Professor Edbroke and Professor Denehy, 

 On behalf of the authorship I would like to thank you both for taking the time to review our paper and for your constructive feedback. Please see below our response to your comments. 

 1. **Despite the justification references used for the sample size, I am not convinced that n=12 would provide enough patients to be representative of the target study population for using telehealth rehabilitation methods.**

**The authors should remove the statement on page 9 that….’similar sample sizes have been used in in other rehabilitation trials in cancer’ This is not a scientific justification of sample size. Thabane et al. 2010 in A tutorial on pilot studies: what, why and how provide a simple confidence interval approach to estimating samples to establish feasibility1.**

**Additionally, I don’t believe that 12 patients will provide enough information on efficacy and variance estimates for the battery of outcome measures to estimate treatment effects in future trials and may lead to biased or unrealistic estimates.**

**Further, the authors should keep in mind the impact that the COVID-19 pandemic could potentially have on the outcomes collected, including CPET, physical activity levels and health-related quality of life and caution against the use of these data for powering future trials.**

*Response:*

 Thank you for your thorough and helpful feedback.

 The statement on page 9 has been removed.

 We have carefully considered your comments regarding the sample size. We acknowledge the sample size is small in comparison to many other feasibility studies. The target study population is wide, covering a variety of ages, genders, levels of impairments and socio-economic backgrounds. It will certainly be difficult to fully represent this population in a feasibility study, and our randomised control trials in this population will more effectively reflect the broader population. The characteristics of the sample as a reflection of the target population will be discussed in the manuscript for publication and will be acknowledged as a limitation of it is under-representative of the target population.

  We wish to emphasise that the primary aim of this study is to examine the feasibility of implementing the ReStOre programme virtually using video-conferencing software. Therefore, the main aspects of feasibility we will explore are related to how the programme translates to a virtual rehabilitation setting. We believe a sample size of 12 will provide sufficient data for the feasibility outcomes of recruitment, adherence, acceptability, retention and adverse incidents, as well as qualitative interviews and the telehealth usability questionnaire. 

 •    No minimum sample size is required for the Telehealth Usability Questionnaire (Parmanto et al. 2016). 

 •    In interview-based studies, data saturation can be achieved with 12 interviews (Guest et al. 2006). In accordance with recommendations from Fusch et al. (2015), we will apply specific measures to ensure the best chance of reaching data saturation: use of saturation grid, two-party coding of transcripts, and suitably designed interview guides.

 As described in the paper’s introduction, the feasibility and effectiveness of the ReStOre programme has already been evaluated in an in-person setting, with both a feasibility study and a pilot randomised controlled trial. Regarding the information needed to estimate treatment effects in future trials, the pilot study and RCT with the ReStOre programme will assist greatly in providing this information, and it will not solely be acquired from this feasibility study. To communicate more clearly that the secondary outcomes are exploratory only and we do not aim to derive treatment effects from this data, we have included the following statement has been in the ‘Outcomes’ section:

 “These outcomes are exploratory only as the sample size is not sufficient to demonstrate treatment effect.”

 As you have highlighted, the secondary outcomes of CPET, physical activity levels and health-related quality of life may be influenced by the government-enforced restrictions, personal decisions to ‘cocoon’ and subsequently reduce physical activity, or indeed by the effects of the disease itself. To understand the effects of the COVID-19 pandemic on participants, we will include the following question on the interview guide:

 “How do you feel your health, fitness and overall wellbeing has been impacted by the COVID-19 pandemic and the restrictions imposed over the last year?”

 In addition, the potential influence of Covid-19 restrictions on secondary outcomes for this study will be highlighted as limitations in future manuscripts detailing the study results.

**2.This is a sub study of the larger RESTORE RCT. It is not documented how recruitment will work alongside the larger RCT at the one hospital site? This is important to ensure that bias is not introduced if patients have a choice or are not sequentially offered to participate in this smaller virtual sub study. Please add.**

*Response:*

 The main RCT is currently on hold due to the COVID-19 pandemic. Ireland continues to experience high numbers of cases and exercising in groups has not been permitted for over one year. Therefore, recruitment for both trials will not be happening at the same time. We aim to commence recruitment for ReStOre@Home in May 2021 and for ReStOre II after the summer, when, hopefully, the wider population is vaccinated and a return to group exercise is permitted.

 The following statement has been included in the ‘Study Participants’ section:

 “Recruitment for ReStOre@Home will not occur at the same time as recruitment for the main ReStOre RCT.”

**3.   What cut-points will be used for the proportion of eligible patients recruited, adherence to intervention sessions and outcome assessments to guide whether implementing the intervention is feasible? Please add to aims/hypotheses.**

*Response:*

 Cut-points will be as follows:

- Proportion of eligible patients recruited: 50%
- Adherence to intervention sessions: mean of 80% adherence to supervised sessions, 70% adherence to unsupervised sessions.
- Attendance of assessment sessions (expressed in paper as ‘retention’): 100% attendance at T0, 83% attendance at T1

The following statement has been included in the Outcomes section:

 “Feasibility to proceed to a definitive trial of ReStOre@Home will be determined by considering all the above factors, and the specific achievement of the following criteria: ≥50% of eligible patients recruited; mean of ≥80% adherence to supervised exercise sessions and ≥70% adherence to unsupervised sessions; ≥83% attendance at T1 assessment.”

**4.    If the baseline SPPB indicates balance impairments will participants still be prescribed outdoor walking or is loan of a stationary cycle for aerobic exercise possible? Please add to method.**

**If researchers find that a participant has a balance impairment which would make it unsafe to walk outdoors, they will not be included in the study. By clearly identifying in the participant information leaflet and in recruitment discussions that a core component of this study is outdoors walking, we think it is unlikely that a participant will be excluded at assessment for this reason.**

*Response:*

 The following statement has been added to the ‘Study Participants’ section:

 “If there are any findings at assessment which indicate that a person is unsafe to exercise, they will not proceed with the intervention phase of the study.”

**5.    Will participants be provided with equipment (bike/treadmill) in the event that walking outdoors is limited by government COVID-19 restrictions? Our recent experience is that participants may be wary of walking outdoors given the current situation. A suggestion.**

*Response:*

 Walking outdoors has been permitted in Ireland throughout the pandemic, and has even been encouraged by the government, with city and county councils providing specific hours for vulnerable individuals to have preferential use of local parks. We will discuss participant’s levels of comfort around walking outdoors and provide safety advice such as wearing a mask and avoiding exercising outdoor in times where parks and pavements are busy. Unfortunately, we do not currently have the means to loan home exercise equipment. 

**6.    The authors state one of the key aims of the programme is to increase participants’ self-efficacy over their recovery – the authors could consider including a patient-reported self-efficacy questionnaire to assess this. It would also strengthen the study to have an objective measure of physical activity using accelerometry, in addition to the use of the Godin Leisure Time Exercise questionnaire. A suggestion.**

*Response:*

 Thank you for these two helpful suggestions. We aim to examine changes in self-efficacy through the qualitative interviews, however on reflection we can see that the study will benefit from this being addressed more explicitly. We have therefore added the following questions to the interview guide, which directly explore self-efficacy concepts: 

 “Have you noticed any changes in your confidence, specifically around being able to do the things that are important to you? How do you feel now about starting or completing something (a task or personal goal) that you find challenging?”

 We have chosen not to use an accelerometer because, as physical activity is not a main outcome of the study, objectively measuring physical activity is not a priority in this feasibility trial. Additionally, as participants will be using videoconferencing software as well as the heart rate monitor and its associated smartphone app, we feel this is already a lot of novel technologies for this population and do not wish to over-burden them from a technological perspective. The results from this study will inform the feasibility of using additional wearable technology in future studies.

**7.    The authors state and Figure 2 highlights that this is a complex intervention. Will weekly video calls from the dietitian, the physiotherapist and group education sessions be co-ordinated/performed in conjunction to reduce the burden on participants of multiple weekly contacts? Please add to method.**

*Response:*

 Video calls and sessions will be scheduled to suit participant’s personal commitments, and, as much as possible, will be grouped together and scheduled at the same time each week. This will be achieved as follows: at recruitment, researchers will ask participants what times and days suit best for attending sessions online and, with this information, a 12-week timetable will be generated for each participant and provided at the start of the intervention. Group sessions (strength training, education) will be at the same time each week. We aim to run 1:1 sessions directly before or after group sessions as able, but this will not always be possible due to staffing numbers. Participants can alter 1:1 sessions on the timetable by consulting the study physiotherapist or dietician as needed.

 The following statement has been added to the ‘Intervention’ section:

 “To ensure the schedule is acceptable to each participant, group sessions will be planned for the same time each week and 1:1 sessions will be scheduled at times chosen by participants.”

**8.    It is not clear (or may have missed it) if all outcome measures are being performed at the hospital? Clearly CPET would be but for others there is potential to undertake these remotely as well? This should be made clear.**

*Response:*

 This is detailed in the ‘Study Participants’ section as follows:

 “In advance of the assessment as much information as possible will be collected via telephone interview e.g. background medical history, dietary interview and questionnaires will be provided in advance to minimise face to face contact.”

 We hope that you find the above changes to the manuscript satisfactory. 

 Kindest regards, 

 Dr Linda O'Neill
